# Administration of a novel penicillamine-bound membrane: a preventive and therapeutic treatment for abdominal adhesions

**DOI:** 10.1186/1471-2482-11-5

**Published:** 2011-02-25

**Authors:** Qiang-Ye Zhang, Sheng Ma, Dong Xi, Wen-Tong Zhang, Ai-Wu Li

**Affiliations:** 1 Department of Pediatric Surgery, Qilu Hospital, Shandong University, 107 Wenhuaxi Road, Jinan, Shandong, 250012, China; 2 Qingzhou Clinical College, Weifang Medical University, China; 3 Department of Pediatrics, The Children's Hospital of Philadelphia, 34thStreet and Civic Center Boulevard, Philadelphia, Pennsylvania, 19104, USA

## Abstract

**Background:**

Adhesions formation is a significant postsurgical complication. At present, there is no effective method for preventing adhesions formation [1], although barrier products such as Dextran (Dex) [2] and sodium hyaluronate (SH) [3] have proved the most clinically successful [4-6], This study is designed to investigate the preventive and therapeutic potential of a novel penicillamine-bound membrane for abdominal adhesions formation.

**Methods:**

150 rats were involved in the present study. All animals were randomly divided into 6 groups (1 vehicle group and 5 test groups respectively treated with dextran, sodium hyaluronate, penicillamine, penicillamine-bound membrane or non-penicillamine-bound membrane). The occurrence, grade and score of abdominal adhesions were compared between the different groups. The breaking strength of incision was compared between the vehicle group and the penicillamine, membrane with/without penicillamine - treated groups. Expression of collagen type I was compared between the vehicle and penicillamine-treated group. The occurrence of adhesions was compared between the Dextran (Dex), sodium hyaluronate (SH), penicillamine-treated group and membrane with or without penicillamine- treated groups.

**Results:**

Penicillamine and penicillamine-bound membrane had significant preventive effects on abdominal adhesions formation, better than dextran, sodium hyaluronate and non-penicillamine-bound membrane. However, neither of them influenced incision healing, although they insignificantly decreased the breaking strength of the incision. Penicillamine-bound membrane, which can be loaded locally and more efficaciously, shows greater advantages than penicillamine.

**Conclusions:**

Penicillamine-bound membrane can be applied as an effective therapeutic intervention for abdominal adhesions with inconsequential side effects.

## Background

Adhesions develop in over 90% of patients after abdominal operations [3,7] and can lead to significant postsurgical complications, including small bowel obstruction, infertility, chronic pelvic pain and difficult re-operative surgeries [2,8]. Adhesions formation is a dynamic and complex process, which involves a cascade of reactions of cellular, biochemical, immunological and biomechanical factors [9]. Unfortunately, there is no available marker to predict the occurrence or severity of adhesions preoperatively [10] and therapeutic prevention still remains a challenge.

At present, the prevention of adhesions formation after surgery has focused on minimizing peritoneal trauma and reducing the implantation of foreign materials into the peritoneal cavity, as they may aggravate the inflammatory response [11-14]. Numerous approaches have been attempted, including profibrinolytic agents and physical barriers [3,15], such as Dextran (Dex) [2] and sodium hyaluronate (SH)[3]. While the barrier products have been proven the most clinically successful [4-6], there is no effective method of preventing adhesions formation currently [1]

Previously, penicillamine was reported to prevent collagen fibers from crossing into non-soluble collagen tissue and inhibit the maturation of dissoluble collagen. Recent studies indicated the possibility of oral D-penicillamine-induced prevention on peritoneal adhesions band formation [16-18]. We therefore hypothesized that it can prevent the fibrin from converting into permanent fiber adhesions tissue. Hereby, we developed a novel membrane, which is composed of two regents- penicillamine and hyaluronic acid, and then applied this penicillamine-bound membrane to treat abdominal adhesions in an animal model, in order to identify its preventive and therapeutic potential for adhesions formation.

## Methods

### Method for manufacturing novel penicillamine-bound membrane

Chitosan [2](Shanghai Qisheng Biologic Agent Company), or polylactic acid or hyaluronic acid [10](Center for New Drug Evaluation, Shandong University) was individually dissolved into saline at the concentration mentioned in previous literatures. Penicillamine (Catalog number: 000108, Shanghai, PR China) was dissolved into three different solutions. The solutions were drained into the flat bottom plastic container and dried thoroughly until polymerized. The thickness and dissolve time for the three different kinds of polymerized membranes were measured in order to select the best substrate of penicillamine-bound membrane. The release of penicillamine was defined by dissolving the membranes into saline solution. Eventually, hyaluronic acid was chosen for the substrate of penicillamine-bound membrane. Hyaluronic acid and aluminum chloride (at the concentration of 5%) were dissolved into autoclaved PBS to make solution 1. Carboxymethyl Cellulose was dissolved in double-distilled water (ddH_2_O) to make solution 2. And then solution 1 and 2 were 1:1 mixed thoroughly. 2.5 ml of 10% penicillamine was pipetted into 50 ml mixed solution in order to lead to cross-linking between penicillamine and substrate. The solutions were drained into the flat bottom plastic container and dried thoroughly for 4-7 days until fully polymerized in air. The thickness of penicillamine-bound membrane was about 0.1 mm, and the degradation of the membrane cost 5 days in corpore. We tested the concentration of penicillamine of the solution after the membrane was dissolved into saline at a different time.

### Animal model of abdominal adhesions

Total 150 rats (Wistar rats of both genders from animals facility of Shandong University) at 9 weeks of age, weighing 200~230 g, were involved in the present study with 25 animals per group in order to calculate the occurrence of abdominal adhesions. The rats were treated under the animal use guidelines of Institutional Animal Care and Use Committee (IACUC) at Qilu Hospital & College of Medicine, Shandong University. Rats were allowed to adapt to the new environment for 1-2 days prior to experimental study. The study was approved by the ethics committee of Qilu Hospital, Shandong University.

**Figure 1:**
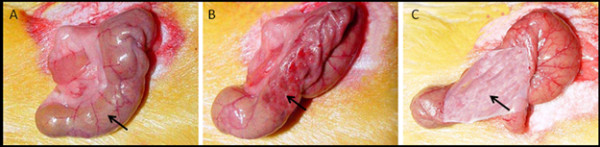
**Animal model of abdominal adhesion**. Arrow in (A) shows the caecum of normal animals. (B) Shows that the caecum is scratched with dry gauze at 2 cm × 2 cm, which is treated as the sham group. While the arrow in (C) shows that, the scratched caecum is covered with penicillamine-bound membrane.

All animals were randomly divided into six groups (25 per group), including the vehicle group (A) and five treated groups (B, C, D, E and F). All groups were anesthetized with 10% chloral hydrate at the dosage of 300~350 mg/kg by intraperitoneal injection, and then underwent abdominal surgery through midline incision 1.5 cm in length. The caecum serosa was scratched with dry gauze at 2 cm × 2 cm [19] (Figure 1). One milliliter saline was put into the rats' abdominal cavity in group A, while 1 ml 40% Dextran (Dex), 0.5 ml sodium hyaluronate (SH) (Shandong Zhengda Freda Tragacanth Company) and 1 ml 3% penicillamine for group B, C and D. The scratched area on caecum serosa in group E was covered by penicillamine-bound membrane (Figure 1), and group F was covered by non-penicillamine-bound membrane. The membrane was not fixed and adhered to the scratched area naturally.

### Tissue preparation

Half of the animals in each group (about 10 animals for every group and time point) were sacrificed at post-surgical day 7, while another half was sacrificed at post-surgical day 14. The adhesions tissue inside the abdominal cavity in different groups was removed and stored in 4% paraformalhyde, then subjected to immunohistochemistry staining. The incision, associated with the lateral skin tissue in different groups, was sheared at 4×0.5 cm to test its breaking strength.

### Measurement of adhesions degree and breaking strength of incision

The occurrence of adhesions was calculated as the ratio between animals with adhesions tissue and total animals within that group (Table 1). The adhesions grade and score in different groups were defined by Bigatti's method [20](Table 2). The breaking strength of incision was measured by a strength-tester (See Additional file 1). After removing the stitches from the incision tissue, it was connected with a water container by a pulley and the breaking strength was defined by the gravity of the water (The Unit was gram), which was drained into the container when the incision tissue was broken. All of these measurements were performed by a blinded observer.

**Table 1:** Comparison of the occurrence of adhesion, adhesion score and breaking strength of incision between control (A) and treated groups (B, C, D).

Group (s)	Occurrence of adhesion		Adhesion score		Breaking strength of incision(unit: g)	
	
	Postsurgical day 7	Postsurgical day14	Postsurgical day 7	Postsurgical day14	Postsurgical day 7	Postsurgical day14
Control (A)	100%	100%	7.625 ± 2.92	9.25 ± 1.91	245.1 ± 16.51	323.13 ± 43.77

Dextran (B)	48%	80%	2.9 ± 1.19*	4.625 ± 2.92	100.6 ± 20.02*	190.45 ± 38.33*

SH (C)	56%	80%	3.5 ± 1.6*	5.25 ± 1.91	115.0 ± 15.5*	183.6 ± 20.0*

Penicillamine (D)	40%	48%	2.7 ± 3.19*	2.5 ± 1.82*	198.0 ± 12.35	287.8 ± 11.09

(* means p < 0.05, compared to control level)

**Table 2:** Adhesion Score (Bigatti's method):

Characteristic	Adhesion Score
Tenacity	
None	0
Adhesions essentially fell apart	1
Adhesions lysed with traction	2
Adhesions required sharp dissection	3
Type	
None	0
Filmy, no vessels (transparent)	1
Dense, no vessels (translucent)	2
Dense, vascular, small vessels (diameter 50 μm)	3
Dense, vascular, large vessels (diameter 50-110 μm)	4
Extent (% of SILASTIC patch surface covered by adhesions)	
0	0
< 25	1
25-50	2
50-75	3
> 75	4

### Immunohistochemistry

Slices (40 μm) were made from 4% paraformalhyde-fixed adhesions tissue with a microtome, then transferred into 0.1 M phosphate buffer (PB) (pH = 7.4). The slices were incubated in 1:500-diluted polyclonal rabbit anti-collagen type I (Beijing Biosynthesis Biotechnology Co., Ltd., China) at 4°C overnight, and then washed in 0.1 M PB three times. Slices were then transferred into avidin-biotin-peroxidase complex and incubated for 20 min at 37°C, then washed with 0.1 M PB, incubated with 3, 3-diaminobenzidine (DAB) tetrahydrochloride for 5-15 min and washed three times in 0.1 M PB. Slices were then mounted onto gelatin-treated slides, dried overnight, and dehydrated serially with 50%, 70%, 95% ethanol once, and 100% ethanol and xylene twice. Slides were then coverslipped using the mounting solution and viewed under the microscope. Negative control experiment was performed by applying 0.1 M PB solution as the primary antibody.

### Data analysis and statistics

The occurrence of adhesion was compared by Chi-square test. The adhesion score, the breaking strength of incision were compared between different groups by one-way ANOVA with post hoc Tukey's test. Data was shown as percentage or mean ± SD. *P*< 0.05 was considered statistically significant. Data analyses were performed using SPSS statistical program version 16.0.

## Results

### Penicillamine prevents the abdominal adhesions formation significantly

An animal model of abdominal adhesions was achieved with a success rate of 88.33%. About 24 animals in total died during surgery, about 4 animals per group for reasons such as bleeding or overdose of anesthesia. Animal death occurred across all groups, which could indicate no potential toxicity of any of the compounds used. Dead animals were discarded from our study. Additionally, our experiments showed no significant difference in adhesion occurrence and scores relating to the sex of the animal.

**Figure 2:**
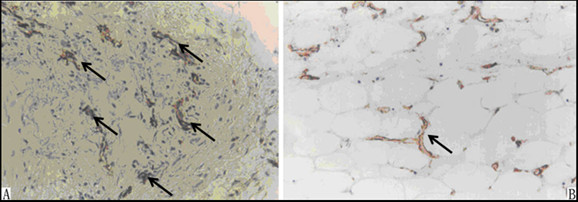
**Comparison of adhesion formation in different groups by immunochemistry staining**. Arrows in (A) and (B) show the stained collagen I. Significantly less collagen I and fibers are found in penicillamine-treated group (B) than in the sham group (A).

**Figure 3:**
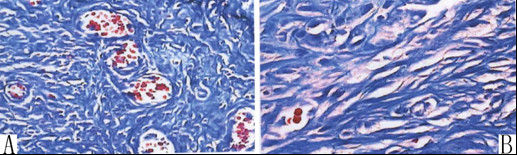
**Comparison of wound healing in different groups by immunochemistry staining**. Blood vessels hyperplasia is observed in the sham group (A), while not in the penicillamine-treated group (B).

The occurrence of adhesions in group A and D was summarized in Table 1. Compared to the control group, the occurrence in group D was significantly lower at postsurgical day 7 and 14 (P = 0.0326, P < 0.05). Further, the adhesions score in group D was significantly decreased 7 days or 14 days after the surgery, compared to control level (P = 0.0064, P < 0.01). Immunohistochemical staining showed that more collagen fibers (Figure 2) and blood vessel hyperplasia (Figure 3) were observed in group A than in group D (Figure 2 at 10×, and Figure 3 at 40×magnification).

### Penicillamine has more preventive effects on abdominal adhesions than dextran (Dex) and sodium hyaluronate (SH)

Compared to group B and C, penicillamine decreased the adhesions score in group D most significantly (P = 0.0326, P < 0.05). The occurrence of adhesions in group D was significantly lower at postsurgical day 7 (40% for D, 48% for B, 56% for C) and day 14 (48% for D, 80% for B, 80% for C).

### Penicillamine-bound membrane shows greater benefits in therapeutic prevention of abdominal adhesions than penicillamine

The carrier for the penicillamine must be stable, non-toxic, non-irritating and not react with the drug. The Chitosan dissolve in acid solution, which has irritation. Nevertheless, the polylactic acid dissolve in organic solvents (chloroform, acetone, e.g.) in which penicillamine can't dissolve. Penicillamine-bound membrane was developed by using hyaluronic acid as the substrate. The membrane was made by only natural hyaluronic acid, which dissolved into the saline in 15 minutes, and so penicillamine was released thoroughly from the membrane. The new membrane was made by hyaluronic acid, aluminum chloride and carboxymethyl cellulose, which dissolved into the saline in 5 days. Penicillamine has a more prolonged period of action.

**Figure 4:**
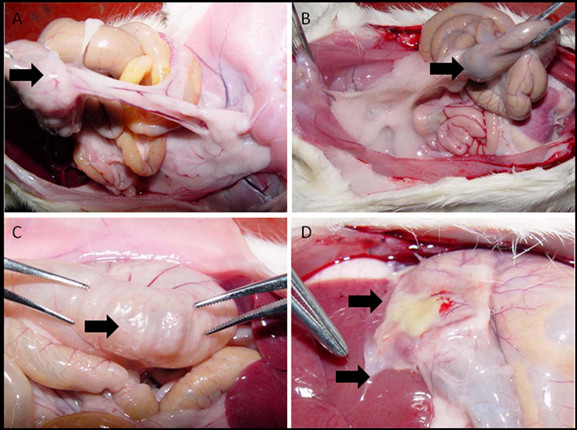
**Comparison of adhesion formation in the sham and penicillamine-bound membrane-treated groups**. Arrows in (A) and (B) show that abdominal adhesion tissue is found in the sham group, while the adhesion is not found in the penicillamine-bound membrane- treated group(C) and (D). Arrows in (C) and (D) show that the scratched caecum is recovered.

The concentration of penicillamine in the membrane is 1.501 ± 0.023 mg/cm^2^. The occurrence of abdominal adhesions in the penicillamine-bound membrane-treated group (40%) was significantly lower than control (100%), penicillamine-treated groups (48%) and non-penicillamine-treated groups (78%) (Table 1) on postsurgical day 14. Comparison of adhesions score (Table 1) showed a significance between control and treated groups, indicating that both penicillamine and penicillamine-bound membrane successfully prevented abdominal adhesions formation, which was confirmed by morphological observation (Figure 4). Moreover, penicillamine-bound membrane showed better effects than penicillamine itself and non-penicillamine-bound membrane (P = 0.0046, P < 0.01) (Table 3). Penicillamine-bound membrane could be loaded directly and locally onto the traumatic area, contributing to its advantages in clinical administration than penicillamine.

**Table 3:** Comparison of occurrence of adhesion, adhesion score and breaking strength of incision between membranes with/without penicillamine- treated groups.

Group (s)	Occurrence of adhesion		Adhesion score		Breaking strength of incision(unit: g)	
	
	Postsurgical day 7	Postsurgical day14	Postsurgical day 7	Postsurgical day14	Postsurgical day 7	Postsurgical day14
Penicillamine -bound Membrane (E)	40%	40%	1.7 ± 1.45*	2.0 ± 1.6*	228.6 ± 19.97	331.7 ± 15.17

Non-Penicillamine -bound Membrane (F)	80%	92%	7.1 ± 1.3	8.91 ± 2.32	259.4 ± 18.32	376.4 ± 23.43

(* means p < 0.05, compared to non-penicillamine -bound membrane- treated group)

### Penicillamine and penicillamine-bound membrane did not influence the incision healing, although they insignificantly decreased the breaking strength of incision

Incision healing occurred very well in all groups. Compared to postsurgical day 7, the breaking strength of incision of each group was higher at postsurgical day 14. Compared to group A, the breaking strength of incision in group D, E and F were lower at postsurgical day 7 or day 14. The possibility exists that tensile strength of the abdominal wound might have been more affected if the membrane had been placed immediately deep to the laparotomy incision. Penicillamine and penicillamine-bound membrane did not influence the incision healing, although they insignificantly decreased the breaking strength of incision (Table 1, 3) (P > 0.05).

## Discussion

Postsurgical abdominal adhesions have a great impact on the quality of life of millions of people worldwide. Small bowel obstruction and others complications of adhesions are serious, causing not only morbidity but also mortality [8,21]. Adhesions are non-anatomic connections of fibrous tissue within normal peritoneal surfaces [7]. It may have a potential benefit, including neovascularization of ischaemic structures such anastomoses, but it is also responsible for various clinical problems [22].

The abdominal formation of fibrin is a common pathophysiological pathway for adhesions. Fibrin is formed after peritoneal injury, which can cause fibrinous adhesion. If the fibrinolytic system, which results in lysis of abdominal fibrin, is not activated, the adhesions will become fibrous [23]. This can be explained when the equilibrium between coagulation and fibrinolysis is disturbed [24-26]. Our present study confirmed this by the evidence of more collagen I fibers observed in the abdominal adhesions animal model than in the vehicle group.

Apart from the formation of fibrin, a complex interaction of biochemical components, including inflammation, fibrinolysis and wound healing, is involved in the pathological process of abdominal adhesions [27]. For instance, initially the deposition of fibrin is regulated and maintained by growth factors and cytokines [28]. After the first week and up to a month, the matrix is remodeled and replaced by persistent proteins, such as collagen, and revascularization occurs. Fibrinolysis stimulators, such as tissue plasminogen factor (t-PA), and urokinase and fibrinolysis inhibitors, such as plasminogen activator inhibitor type I (PAI-1), transforming growth factor (TGF)-β, a key molecular mediator of pathological fibrosis, have also been shown to play a role in adhesions pathogenesis [29,30]. The interrelationship between all the factors remains largely unknown, therefore, identifying the effective treatment or prevention for abdominal adhesions remains a big challenge.

Numerous approaches have been used to prevent adhesions [15]. The three main principle pathways are: (1) decreasing the trauma to the peritoneum; (2) medical intervention in the fibrin formation/degradation balance, and (3) barriers (including fluid barrier and membranes) preventing organs from bridging over to other structures in the abdomen and thereby forming adhesions. Unfortunately none of these measures have proven uniformly effective under all surgical conditions. Barrier products, including hyaluronic acid-carboxymethyl cellulose membrane have been the most clinically successful in reducing adhesions formation by preventing the close apposition of injured tissues. However, treatment with these products induced many side effects, such as postponing wound healing. Furthermore, many treated models have a high standard deviation, which makes the relevance of results with only moderate effects questionable.

Penicillamine can decrease the permeability of vessels by inhibiting aggregation of platelets, stabilizing lysosome and inhibiting releasing of lysomal enzymes. It can also attenuate immune reaction and decrease blood disk effusion and fibrin deposition by inhibiting generation of IgG and IgM and decreasing antigen-antibody complex in blood-serum, which blocks the first stage of abdominal adhesions. It is reported that D-penicillamine administration markedly reduces severe adhesions band formation without severe side effects [18]. Therefore, we hypothesized, based on these results, that a combination of penicillamine and barrier products may be a better treatment for adhesions.

In the present study, we developed a novel penicillamine- bound membrane, which used hyaluronic acid as the ideal substrate, and then found that both penicillamine and penicillamine-bound membrane have better therapeutic effects on preventing abdominal adhesions than Dextran (Dex), sodium hyaluronate (SH) and non-penicillamine-bound membrane. Both of them did not affect wound healing. Although they decreased the breaking strength of incision insignificantly at postsurgical day 7 but, this decrease was ameliorated at postsurgical day 14. Penicillamine-bound membrane showed greater benefits than penicillamine itself in preventive effects and local administration. Recent studies investigated that penicillamine can inhibit blood vessel hyperplasia[31], which plays an important role in adhesions generation, by inhibiting the proliferation of endangium and smooth muscle cell, and this was confirmed by our results.

## Conclusions

The present research indicated that penicillamine-bound membrane can be applied as an effective therapeutic intervention for abdominal adhesion with inconsequential side effects, but further studies on the detailed mechanisms for treating abdominal adhesion are still warranted.

## Competing interests

The authors declare that they have no competing interests.

## Authors' contributions

Q-YZ carried out the preparation of penicillamine-bound membrane, created the animal model of abdominal adhesions and drafted the manuscript. SM carried out the tissue preparation and measurement of adhesions degree and breaking strength of incision. DX carried out the Immunohistochemistry. W-TZ participated in the design of the study and performed the statistical analysis. A-WL conceived the study, and participated in its design and coordination.

All authors read and approved the final manuscript.

## Pre-publication history

The pre-publication history for this paper can be accessed here:

http://www.biomedcentral.com/1471-2482/11/5/prepub

## Supplementary Material

Additional file 1 **Test for breaking strength of incision**. Step 1. Connecting the incision with an empty water container. Step 2. Draining the water gradually into the container until the incision is broken, then calculating the breaking strength by this formula (Breaking strength = the gravity of total water). Click here for file
