# Supplementary figures and images for: Administration of a novel penicillamine-bound membrane: a preventive and therapeutic treatment for abdominal adhesions
Source: BMC Surg. 2011 Feb 25;11:5. doi: 10.1186/1471-2482-11-5 (PMC3053215; doi:10.1186/1471-2482-11-5)

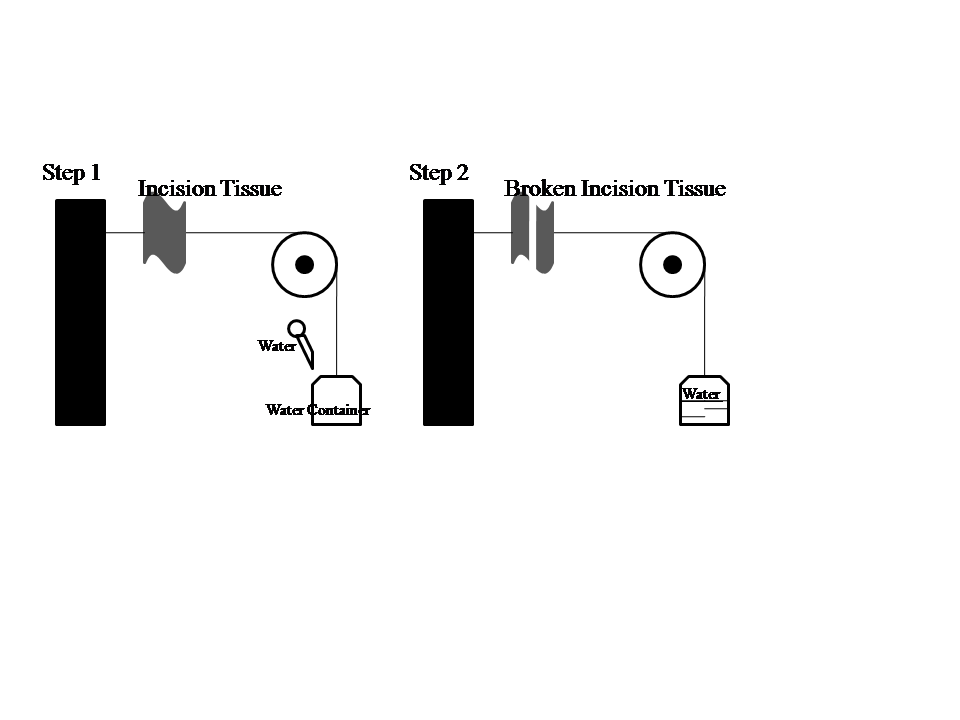

Supplement: Additional file 1 — Test for breaking strength of incision. Step 1. Connecting the incision with an empty water container. Step 2. Draining the water gradually into the container until the incision is broken, then calculating the breaking strength by this formula (Breaking strength = the gravity of total water). [file 1471-2482-11-5-S1.tiff]
